# Heme Oxygenase-1 (HMOX1) Gene Polymorphisms, Thrombosis and COVID-19: Correspondence

**DOI:** 10.31661/gmj.v12i.2952

**Published:** 2023-06-14

**Authors:** Rujittika Mungmunpuntipantip, Viroj Wiwanitkit

**Affiliations:** ^1^ Private Academic Consultant, 111 Bangkok 122 Bangkok 103300 Thailand; ^2^ Chandigarh University, Punjab, India; Adjunct professor, Joesph Ayobabalola University, Ikeji-Arakeji, Nigeria

**Keywords:** Heme oxygenase-1, Thrombosis, COVID-19

## Dear editor,

we would like to share ideas on the publication “Heme Oxygenase-1 (HMOX1) Gene Polymorphisms as Predictive Markers of Increased Risk of Thrombosis among Patients with Coronavirus disease 2019 (COVID-19) [1].” According to Shakir Mohammed et al., identifying patients at high risk for HMOX-1 pathway activation and thrombosis as well as determining the relationship between HMOX-1 promoter polymorphisms and disease severity and increased risk of thrombosis among COVID19 black patients may be helpful in developing a treatment plan to prevent COVID-19 complications. The hypothesis that HMOX-1 pathway activation and thrombosis are connected to greater morbidity in blacks is presented in the publication by Shakir Mohammed et al. We disagree with them. This article’s discussion of inherited traits may or may not be relevant. We both agree that the genetic component under investigation may be connected to the desired result. The severity of COVID-19 is, however, correlated with a number of genetic differences, such as TMPRSS2, interleukin 1B, TMPRSS2, and HLA polymorphisms [2],[3],[4],[5]. Also, there’s a probability that the current asymptomatic COVID-19 is related to a former clinical manifestation of the illness. The consequences of unanticipated, potentially puzzling genetic alterations should be the focus of future research. In conclusion, Shakir Mohammed et al.’s study on a single genetic variant and conclusion for the interrelationship is still too preliminary, and there may be confounders that can contribute to the severity. According to Shakir Mohammed et al.’s report, HMOX-1 gene polymorphism in blacks may be one of the causes, but it could also be due to other uninvestigated genetic factors, as previously mentioned. As evidence, in an experimental study in which the HMOX-1 gene polymorphism was evaluated for its relationship with disease severity, it was discovered that there is also an impact from other important genetic polymorphisms such as NRF2, NQO1, and MT at the same time [6]. As a result, Shakir Mohammed et al.’s study on a single genetic variant and conclusion for the interrelationship is still too preliminary, and there may be confounders that cause the conclusion to be invalid. A study on a single genetic variant and then drawing conclusions about the association is a common pitfall if the potential effect of other genetic variants is not considered.

## Conflict of Interest

None.
